# Predictive modeling of eye lens dose in interventional radiology: a polynomial regression approach to cumulative fluoroscopy dose

**DOI:** 10.3389/fpubh.2025.1547101

**Published:** 2025-05-20

**Authors:** Mengyun Wu, Fang Yuan, Yeqing Gu, Jinhan Wang, Lin Lu, Zhi Zeng

**Affiliations:** ^1^Radiology Laboratory, Department of Occupational Health and Radiological Health, Chongqing Center for Disease Control and Prevention, Chongqing, China; ^2^Institute of Radiation Medicine, Chinese Academy of Medical Sciences and Peking Union Medical College, Tianjin, China; ^3^School of Political Science and Public Administration, Southwest University of Political Science and Law, Chongqing, China; ^4^Office of Policy and Planning Research, Chinese Center for Disease Control and Prevention, Beijing, China

**Keywords:** interventional radiology, eye lens dosimetry, cumulative fluoroscopy dose, quadratic polynomial regression, occupational radiation safety

## Abstract

**Objectives:**

This study aimed to investigate the relationship between cumulative fluoroscopy dose and eye lens radiation dose among interventional radiologists, and to develop a predictive model to enhance occupational radiation safety.

**Methods:**

We collected data from interventional radiologists, focusing on cumulative fluoroscopy dose during procedures and corresponding eye lens doses. A quadratic polynomial regression model was developed to assess the non-linear relationship between cumulative fluoroscopy dose and eye lens dose. The study involved the use of machine-generated cumulative dose data and personal eye lens dosimeters.

**Results:**

The quadratic polynomial regression model effectively captured the non-linear relationship for cumulative doses >20 Gy, enabling precise dose prediction at higher exposure levels where cataract risks escalate. However, the model showed limited accuracy for doses ≤ 20 Gy. This model allowed for more precise prediction of eye lens dose, particularly at higher exposure levels where the risks of radiation-induced cataracts increase significantly.

**Conclusions:**

The quadratic polynomial regression model serves as a potentially valuable tool for real-time monitoring in high-exposure scenarios (>20 Gy), supporting radiation safety protocols in clinical practice. Integration into routine hospital systems may enhance radiation protection protocols and inform policy development, aligning occupational dose monitoring practices with international safety standards.

## Introduction

Interventional radiology (IR) has become an essential field in modern medicine, offering minimally invasive procedures for the treatment of complex conditions such as cardiovascular diseases, cancers, and neurological disorders (1). However, the benefits of these techniques come at the cost of substantial radiation exposure, particularly due to the extensive use of fluoroscopic imaging. Healthcare professionals such as interventional radiologists, nurses involved in endoscopic retrograde cholangiopancreatography (ERCP), orthopedic surgeons using fluoroscopy, and echocardiologists performing transcatheter aortic valve implantation (TAVI)—face elevated occupational radiation risks because of their close proximity to the radiation source and the frequency of exposure.

Among the various organs at risk, the eye lens is particularly radiosensitive. Cumulative radiation exposure has been strongly associated with the development of radiation-induced cataracts, even at relatively low dose thresholds (2–6). Recognizing this risk, international organizations such as the International Commission on Radiological Protection (ICRP) have recommended lowering the dose limit for occupational eye lens exposure to 20 mSv per year averaged over 5 years, with no single year exceeding 50 mSv (7, 8).

In China, despite the rapid development of the healthcare sector and the increasing number of interventional procedures performed, the current regulation still sets the annual dose limit for the eye lens at 150 mSv—significantly higher than international recommendations (9). With ~50,000 interventional radiology professionals nationwide, many of whom operate in high-exposure environments, this discrepancy raises substantial concerns about occupational health risks and long-term ocular safety. Moreover, the adoption of protective measures, such as eye lens dosimeters and leaded eyewear, remains inconsistent. Healthcare workers often cite discomfort and concerns over workload impact as reasons for poor compliance, leading to underreporting and insufficient dose monitoring (10, 11).

Compounding these challenges is the relative paucity of comprehensive, real-world exposure data in China. To date, few large-scale studies have systematically assessed the actual radiation doses received by interventional radiology professionals, creating a critical knowledge gap that hampers evidence-based policy development. Addressing this void is imperative to safeguard medical staff and align China's occupational safety standards with evolving international best practices.

In response, this study aims to develop a novel predictive model using polynomial regression to estimate eye lens radiation dose based on cumulative fluoroscopy exposure. By providing a more accurate and practical tool for dose estimation, our goal is to enhance clinical radiation safety protocols and mitigate the risk of radiation-induced cataracts among interventional radiology personnel. Furthermore, this research seeks to bridge existing gaps in routine eye lens dose monitoring practices, offering empirical data that could inform national policy revisions and promote the adoption of internationally harmonized occupational health standards.

## Methods

This cross-sectional study investigated the relationship between cumulative fluoroscopy dose and eye lens dose among interventional radiology professionals in China. The study was conducted across three tertiary hospitals in Chongqing between January and December 2023. The research was structured into four phases to ensure methodological rigor: (1) preparatory activities (January–March), including the development of a standardized surgical registry form and identification of key data points; (2) protocol setup (April–May), involving equipment procurement, dosimeter calibration, and selection of participating hospitals and staff; (3) training and implementation (June–August), with staff training on dosimeter use and data collection protocols, followed by a 30-day monitoring period (July–August) during which 85 participants wore eye lens dosimeters during fluoroscopy procedures; and (4) analysis and validation (September–December), including statistical modeling, external data collection, and model validation. The methodology ensured accurate data collection, robust statistical analysis, and strict quality control.

### Sample size determination

The sample size for this study was determined based on the expected statistical power to detect a meaningful effect size. We aimed to achieve 90% statistical power to detect a correlation coefficient of 0.512 between cumulative fluoroscopy dose and eye lens radiation dose, with a significance level set at 0.05 for a two-tailed test. This estimate was informed by preliminary pilot data. Simulations demonstrated that a sample of 40 participants would yield 90.5% power (95% CI: 0.897–0.914). To account for potential attrition, a 20% increase was applied, resulting in a target sample size of at least 50 participants. Ultimately, 85 professionals were enrolled, ensuring sufficient power and addressing potential variability in real-world clinical settings. Further methodological details are provided in the Supplementary material.

### Study population

A total of 85 interventional radiologists from two general tertiary hospitals and one tertiary cancer hospital were enrolled, providing a representative sample of interventional radiology practices across varied clinical settings. All participants routinely performed fluoroscopy-guided interventions as either primary or secondary operators, with an average workload of ≥10 procedures per week. Ethical approval was granted by the Institutional Review Boards (Approval Number: KY-2023-004-1), and informed consent was obtained from all participants in accordance with the Declaration of Helsinki.

In accordance with international guidelines (12), the monitoring period for occupational radiation exposure was set at 1 month (30 days) from July to August 2023, aligning with the recommended routine monitoring period of 1 month based on staff radiation exposure levels and work types. Participants were equipped with specialized eye lens dosimeters to record radiation exposure, while cumulative fluoroscopy doses were automatically recorded by the fluoroscopy equipment for each procedure. To ensure accurate dosimeter usage, a supervisory protocol was implemented in each catheterization laboratory, with trained physicists or nurses verifying correct dosimeter placement prior to procedures and overseeing standardized post-procedure storage. Notably, participants did not use protective eyewear during the monitoring period, reflecting typical clinical practice in the participating centers and providing an accurate assessment of unshielded occupational exposure.

### Data collection

Data were collected using a standardized procedural registry developed under the Standardization Research Project for Public Health by the China CDC (BZ2023-Q004). The registry captured detailed variables including operator role (primary or secondary), procedure type, surgical technique, protective measures employed, cumulative fluoroscopy dose, fluoroscopy time, and number of exposure frames. After each procedure, a designated staff member recorded the cumulative fluoroscopy dose, which was obtained directly from the fluoroscopy machine and represents the radiation output during the procedure.

### Eye lens dose monitoring

Eye lens thermoluminescence personal dosimeter (model SSCC-3, Beijing Haiyang Bochuang Technology Co., Ltd.), compliant with the *H*_p_(3) measurement standard (ICRU 1992), were used to monitor radiation exposure. Each dosimeter incorporated two lithium fluoride (LiF: Mg, Cu, P) detectors, with one assigned to each eye. The minimum detectable level (MDL) was determined as three times the standard deviation of background radiation measurements, following ICRU Report 95 recommendations (13). The MDL (0.013 mSv) was set to 3σ (σ = 4.3 μSv), which corresponds to an ~50% probability of detection for a dose equal to the MDL under idealized measurement conditions. This statistical definition reflects the theoretical detection threshold and does not account for other sources of uncertainty such as dosimeter calibration error, environmental variation, or inter-device variability. This choice follows ICRP and ISO 11929 standards, emphasizing false-positive control in low-dose environments. While IUPAC's 3.3σ criterion optimizes signal-to-noise ratios in analytical chemistry, radiation dosimetry universally adopts 3σ, ensuring compliance with radiological safety frameworks.

To illustrate the scale of measurement variability, we compared the standard deviation to natural background radiation. Using a global average dose rate of 0.27 μSv/h, this corresponds to ~2.9 days of background exposure. It should be noted that this value includes both external and internal components of natural radiation. The comparison is intended as a conceptual reference only and does not reflect dosimeter calibration conditions.

To ensure accurate measurements, thermoluminescent dosimeter (TLDs) underwent rigorous calibration. N-100 X-ray source was used for energy response calibration of TLDs. The calibration process involved irradiating TLDs with the N-100 X-ray source and measuring their responses to establish an energy response calibration curve. Correction factors derived from this curve were applied to adjust TLD readings. The uncertainty of this reference irradiation, considering factors such as X-ray source stability and environmental conditions, resulted in a final relative uncertainty of Urel = 6.9%. Additionally, the fluoroscopy machine dose displays were validated through National Metrology Institute of China (NMIC)-certified calibration to ensure compliance with national standards.

To account for energy dependence during calibration, dosimeters were irradiated using both a Cs-137 source (662 keV) and an N-100 filtered X-ray beam. The Cs-137 source provided a stable, high-energy reference, while the N-100 beam better represented the lower-energy range encountered in clinical fluoroscopic procedures. The TLDs demonstrated a batch dispersion of ≤ 3%, compliant with IEC 62387:2020 requirements (14). Detailed technical specifications, calibration results, and schematics are provided in Supplementary material, including dosimeter response characteristics, calibration procedures, and placement schematics (Supplementary Table S1, Supplementary Figure S1).

Participants wore dosimeters bilaterally, secured with an elastic headband. Unlike conventional single-eye monitoring (typically of the left eye, which is closer to the radiation source) (15, 16), this study assessed bilateral doses to inform personalized protection strategies if significant lateral differences were observed. At the end of each monitoring period, adhering to the specifications for individual monitoring of occupational external exposure, dosimeters were collected and read using the thermoluminescent dosimeter reader (TLD, model RGD-3D, Beijing Haiyang Bochuang Technology Co., Ltd). The readings were multiplied by the calibration factor to determine the eye lens dose.

The cumulative fluoroscopy dose per operator—automatically recorded in Gy by the fluoroscopy equipment—was correlated with measured eye lens doses. This approach was adopted because occupational dose estimation via machine-reported data (e.g., cumulative air kerma) is widely accepted as a surrogate for staff exposure in interventional settings, particularly when direct personal monitoring (e.g., full-body dosimeters) is logistically challenging. To address low-dose scenarios, eye lens doses below the minimum detectable level (MDL = 0.013 mSv) were conservatively recorded as $\frac{1}{2}$ MDL (0.0065 mSv), following ICRU Report 95 guidelines. Monitoring periods were capped at 3 months to align with national standards (GBZ 130-2020), minimizing environmental interference and storage-related measurement errors.

### Statistical analysis

All statistical analyses were performed using R version 4.3.2 with RStudio. The normality of continuous variables was evaluated using the Kolmogorov–Smirnov test, supplemented by visual inspection of histograms and assessment of skewness and kurtosis (17). Descriptive statistics were calculated, with normally distributed data presented as mean and standard deviation, and non-normally distributed data as median and interquartile range (IQR). Categorical variables were expressed as frequencies and percentages. Pearson's correlation coefficient was employed to assess the relationship between cumulative fluoroscopy dose and eye lens dose. Given prior studies and preliminary data, a positive correlation was hypothesized.

### Model selection

To predict eye lens dose based on cumulative fluoroscopy dose, the relationship between these variables was first visualized using a scatter plot with a smooth fitted curve generated by a generalized additive model (GAM). Subsequently, several regression models were compared—including general linear regression, restricted cubic splines, and quadratic polynomial regression—to identify the optimal predictive model. Model selection criteria included: (1) Goodness-of-Fit, assessed using adjusted *R*^2^, Akaike Information Criterion (AIC), and Bayesian Information Criterion (BIC), with a higher adjusted *R*^2^ and lower AIC/BIC indicating a better fit; (2) Residual Distribution, where residual plots were examined to confirm that model assumptions were met; (3) Prediction Accuracy, evaluated using root mean square error (RMSE) and mean absolute error (MAE), with lower values indicating better performance.

### Sensitivity analysis

To test model robustness, sensitivity analyses were performed by introducing potential confounding variables such as age, years of experience, and use of protective eyewear. These variables were added individually to the regression model to assess their impact on parameter estimates. The results showed no significant changes, confirming the model's stability. Subgroup analyses based on participants' professional roles (e.g., radiologists) further demonstrated model consistency. The correlation between cumulative fluoroscopy dose and eye lens dose remained significant across all subgroups, with correlation coefficients ranging from 0.70 to 0.80, supporting the model's generalizability.

### Quality control

Strict quality control measures were implemented to ensure the accuracy and reliability of dosimeter readings. Eye lens dosimeters were calibrated at both the beginning and end of the study at the Shanghai Institute of Metrology, following established protocols to account for potential sensitivity drift. To standardize dosimeter placement, thermoluminescent dosimeters (TLDs) were affixed to the lateral canthus of each eye using hypoallergenic adhesive tape. Placement was supervised by designated physicists or nurses in each catheterization laboratory to ensure consistency. The devices were regularly inspected for operational integrity throughout the study. Monthly cross-checking of dosimeter readings against procedural logs ensured data completeness, with discrepancies resolved in collaboration with radiology departments. Additionally, a third-party audit of 10% randomly selected dosimeters was conducted, confirming measurement deviations within the acceptable 20% threshold. Collectively, this study employed a rigorous methodology to investigate the relationship between cumulative fluoroscopy dose and eye lens dose among interventional radiology professionals, which carry important implications for occupational health in interventional radiology.

## Results

This section presents the findings of the study, focusing on the relationship between cumulative fluoroscopy dose and eye lens dose among interventional radiology professionals. The analysis includes descriptive statistics, model fitting, and validation to accurately describe the dose-response relationship, in line with international radiation protection standards.

### Participant characteristics

A total of 85 interventional radiology professionals from three hospitals in Chongqing participated in the study: 39 from Hospital A (45.88%), 35 from Hospital B (41.18%), and 11 from Hospital C (12.94%). Of these participants, 66 were primary surgeons (77.65%) and 19 were secondary surgeons (22.35%). The procedures were primarily cardiac vascular interventions (43.53%), followed by cerebral vascular (12.94%) and abdominal interventions (17.65%). Detailed demographic and procedural characteristics are provided in Table 1.

**Table 1:** Descriptive statistics of the study population and procedures.

**Variable**	**Category**	***n***	**%**
Hospital	Hospital A	39	45.88
	Hospital B	35	41.18
	Hospital C	11	12.94
Surgeon position	Primary surgeon	66	77.65
	Secondary surgeon	19	22.35
Procedure type	Cerebral vascular	11	12.94
	Thoracic	10	11.76
	Cardiac vascular	37	43.53
	Peripheral vascular	11	12.94
	Abdominal	15	17.65
	Others	1	1.18
Procedural access	Femoral artery puncture	38	44.71
	Femoral vein puncture	3	3.53
	Brachial artery puncture	2	2.35
	Radial artery puncture	33	38.82
	Jugular vein puncture	1	1.18
	Other procedures	8	9.41

Describes the demographics and procedure types of the interventional radiology professionals.

### Fluoroscopy and eye lens dose distributions

The distribution of cumulative fluoroscopy doses and corresponding eye lens doses exhibited notable variability. The median cumulative fluoroscopy dose was 5.087 Gy (IQR: 1.054–10.095 Gy). The median left eye lens dose was 0.095 mSv (IQR: 0.056–0.423 mSv) and the right eye lens dose was 0.096 mSv (IQR: 0.050–0.349 mSv). Dose data for left and right eyes were analyzed separately to evaluate potential lateralization effects attributable to operator positioning relative to the radiation source. Given that the cumulative fluoroscopy dose did not follow a normal distribution (Kolmogorov–Smirnov test, *p* < 0.05), non-parametric statistical methods were applied to ensure analytical robustness. Specifically, the Wilcoxon signed-rank test was employed to compare paired eye lens doses. The analysis revealed that left-eye doses were significantly higher than right-eye doses in cardiac-vascular procedures (*p* < 0.05), a finding consistent with previous reports attributing this asymmetry to fixed operator positioning during cardiac interventions (16). By contrast, No significant lateral difference was observed in non-cardiac procedures (see Supplementary Table S3).

### Correlation between cumulative fluoroscopy dose and eye lens dose

**Figure 1:**
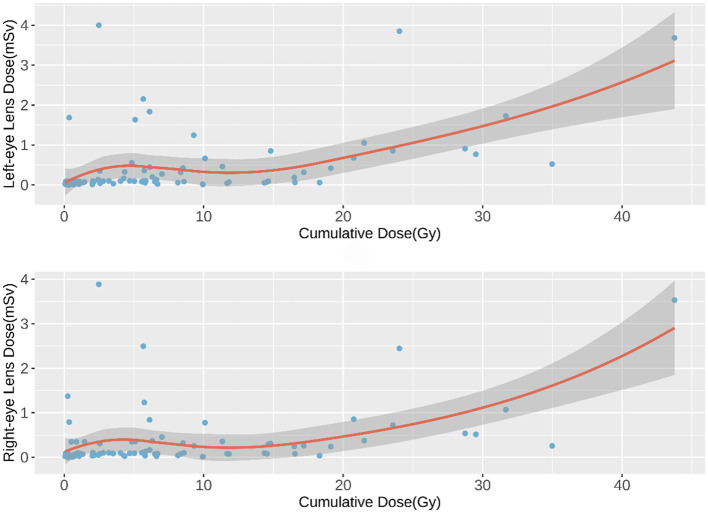
Scatter Plot of Cumulative Fluoroscopy Dose vs. Eye Lens Dose. Visualizes the relationship between cumulative fluoroscopy dose and eye lens dose with a smooth curve.

The relationship between cumulative fluoroscopy dose and eye lens dose was initially explored using a scatter plot with a smooth fitted curve, which suggested a positive, potentially non-linear relationship (Figure 1). To quantify this relationship, a Generalized Additive Model (GAM) was applied, which allowed for non-linearity in the dose-response curve while adjusting for confounding variables such as hospital, surgeon role, procedure type, and access method.

The GAM analysis revealed that cumulative fluoroscopy dose was a significant predictor of both left and right eye lens doses, with *p*-values < 0.001 in both cases. The model explained a substantial portion of the variance, with adjusted *R*^2^ values of 0.367 for the left eye and 0.316 for the right eye. These results are detailed in Table 2.

**Table 2:** Generalized additive model analysis of the relationship between cumulative fluoroscopy dose and eye lens dose.

**Variable**	**Dependent variable**	***F*-value**	***P*-value**	**Adjusted *R*^2^**	**Deviance explained (%)**
Cumulative fluoroscopy dose (Gy)	Left eye lens dose (mSv)	5.492	< 0.001	0.367	50.8
	Right eye lens dose (mSv)	5.439	< 0.001	0.316	46.4

Shows the GAM results, adjusted for confounding variables such as hospital and procedure type.

### Model comparison: general linear, restricted cubic spline, and polynomial regression

To better characterize the non-linear relationship between cumulative fluoroscopy dose and eye lens dose, multiple regression models were compared (Table 3). The general linear regression model provided a baseline fit, but its simplicity limited its explanatory power (adjusted *R*^2^ = 0.255 for the left eye). The restricted cubic spline model, which allowed for more flexible non-linear relationships, performed better, but the best model was the quadratic polynomial regression model.

**Table 3:** Comparison of model performance.

**Model**	**Left eye lens dose (mSv)**			**Right eye lens dose (mSv)**		
	**Linear model**	**RCS model**	**Polynomial model**	**Liner model**	**RCS model**	**Polynomial model**
*P*	**< 0.001**	**< 0.001**	**< 0.001**	**0.028**	**< 0.001**	**< 0.001**
Adjusted *R*^2^	0.255	**0.299**	0.248	0.142	**0.233**	0.201
AIC	193.600	189.122	**183.649**	178.921	170.199	**162.104**
BIC	230.239	228.204	**190.977**	215.560	209.281	**169.432**
RMSE	0.730	0.610	**0.551**	0.629	0.546	**0.540**
*R* ^2^	0.310	**0.416**	0.296	0.229	**0.361**	0.343
MAE	0.515	0.395	**0.347**	0.426	0.346	**0.341**

Adjusted R^2^, AIC, and BIC represent the goodness of fit, while RMSE, R^2^, and MAE represent the prediction accuracy of the models. Adjusted R^2^ values were calculated using the standard adjustment method for the coefficient of determination, accounting for the number of predictors relative to the sample size. P-values < 0.05 indicate that the model fit is acceptable. A smaller AIC, BIC, RMSE, and MAE, and an Adjusted R^2^ closer to 1, indicate better model performance. Bolded values represent the best results for each evaluation metric.

The polynomial regression model not only demonstrated the best statistical fit but also provided better predictive accuracy for clinicians who need to estimate eye lens dose in real time. Its quadratic structure allows for more precise estimation of eye lens dose as cumulative fluoroscopy dose increases, especially at higher exposure levels where the risks of radiation-induced damage accelerate. This makes the polynomial model a practical tool for healthcare professionals, enabling them to identify critical exposure thresholds and implement preventive measures more effectively. In clinical settings, this model can be integrated into routine radiation monitoring, helping to safeguard interventional radiology staff from excessive eye lens exposure.

The quadratic polynomial model demonstrated the best statistical fit among the evaluated models, as evidenced by the lowest AIC and BIC values and the highest prediction accuracy (RMSE = 0.551, MAE = 0.347). However, the model explained only a small portion of the variance in eye lens doses (adjusted *R*^2^ = 0.248 for the left eye, 0.201 for the right eye), indicating that unmeasured factors—such as operator positioning relative to the radiation source, scatter radiation patterns, and procedural complexity—likely contribute substantially to dose variability. These findings align with prior studies emphasizing the multifactorial nature of occupational radiation exposure in interventional settings. The dose-response curves for the left and right eyes were modeled as follows:


(1) $$\begin{array}{l} Left\;eye\;dose:\;Y_{left\;}=0.0013401X^{2}+0.2375347 \end{array}$$

(2) $$\begin{array}{l} Right\;eye\;dose:\;Y_{right\;}=0.0010375X^{2}+0.2080989 \end{array}$$

*X* represents cumulative fluoroscopy dose (Gy), and *Y* denotes predicted eye lens dose (mSv)

**Figure 2:**
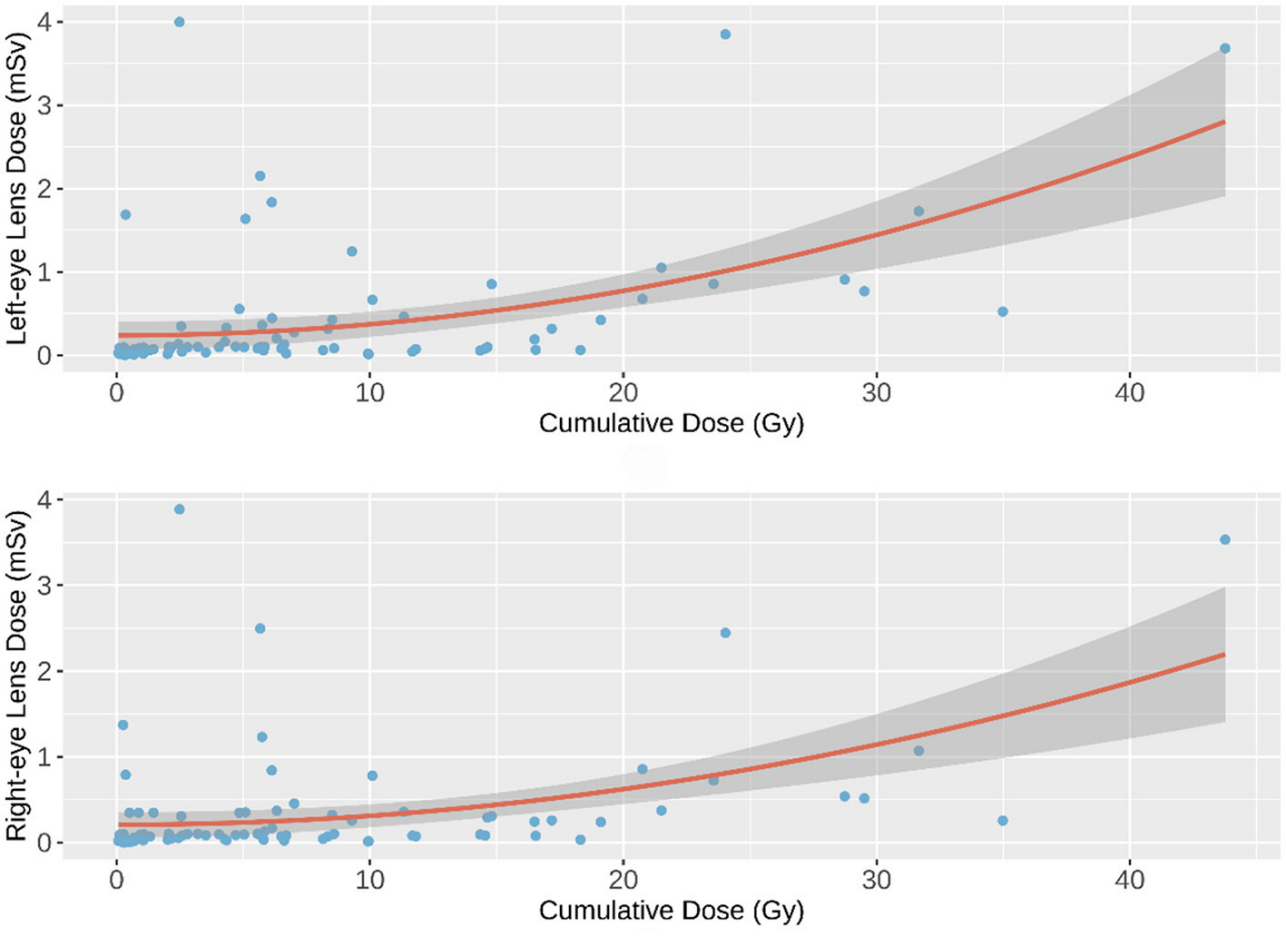
Quadratic Polynomial Regression Model. The quadratic polynomial regression curves (solid lines) depict the predicted eye lens dose as a function of cumulative fluoroscopy dose. The dark gray band indicates the 95% confidence interval (CI) of the model predictions, reflecting uncertainty in the estimated relationship.

Figure 2 illustrates the dose-response curves for the left and right eyes, modeled via quadratic polynomial regression. The dark gray band surrounding each curve represents the 95% confidence interval (CI) of the predicted values. For a detailed comparison of the models, please refer to the Supplementary material.

### Validation of the polynomial regression model

As shown in Figures 1, 2, a weak negative correlation was observed between eye lens dose and cumulative fluoroscopy dose below 20 Gy, indicating limited predictive utility of the model in this range. Piecewise regression analysis, using a threshold of 20 Gy, demonstrated that the quadratic polynomial model was statistically significant for cumulative doses exceeding 20 Gy, but not for doses at or below this threshold. These findings suggest that the model is applicable primarily in high-dose scenarios (>20 Gy).

To validate the polynomial regression model, additional data were collected from a separate hospital, monitoring 28 interventional radiology professionals over a 30-day period. Validation results indicated that 79.6% of left eye lens dose estimates and 75.0% of right eye lens dose estimates fell within the model's 95% confidence interval, thereby confirming the model's reliability for predicting eye lens dose based on cumulative fluoroscopy exposure in high-dose settings.

## Discussion

This study provides new insights into the relationship between cumulative fluoroscopy dose and eye lens radiation dose among interventional radiology professionals in China. By developing and validating a quadratic polynomial regression model, we have offered a novel approach for estimating occupational eye lens dose in high-exposure scenarios, where the risk of radiation-induced cataracts is of particular concern. Our findings contribute valuable empirical evidence that may inform improvements in clinical radiation protection strategies and guide policy development.

### Key findings and implications

We observed a positive correlation between cumulative fluoroscopy dose and eye lens dose. However, our data demonstrated a clear non-linear relationship, particularly at higher cumulative exposure levels (>20 Gy), where eye lens dose increased disproportionately. The quadratic polynomial regression model was selected as the most suitable due to its ability to accurately reflect this non-linear association. These results align with the radiobiological understanding that radiation-induced cataractogenesis exhibits a dose-response relationship with a potential acceleration at higher exposure thresholds.

Our study also highlights the importance of considering lateral asymmetry in eye lens dose exposure. We found that left-eye doses were significantly higher than right-eye doses in cardiac-vascular procedures, consistent with established evidence that the operator's left side is typically closer to the radiation source during these interventions. This finding underscores the need for targeted protective strategies, such as the routine use of leaded eyewear and ceiling-suspended shields, especially for the left eye.

### Practical implications for clinical practice and policy-making

The practical application of this model in clinical settings is straightforward. By incorporating the polynomial regression model into dose-monitoring systems, hospitals can enhance real-time prediction of eye lens doses, particularly in high-exposure environments. This enables clinicians to proactively implement protective measures, such as adjusting procedural techniques or increasing the use of leaded eyewear, before exposure reaches harmful levels.

The quadratic polynomial regression model developed in this study offers significant advancements in occupational radiation safety. It provides healthcare professionals with an effective tool for ongoing dose management, supporting timely decision-making and promoting adherence to safety standards. When embedded into hospital systems, the model can automatically trigger alerts as cumulative exposure approaches or exceeds predefined limits, facilitating immediate intervention. It is important to emphasize that the quadratic polynomial model provides a statistical approximation of the observed non-linear trend, particularly at higher exposure levels. The improved fit over a linear model (as shown by BIC) reflects the mathematical characteristics of the dataset rather than a mechanistic explanation of radiation-induced lens damage. As such, while the model is useful for practical dose monitoring, it should be interpreted with caution in mechanistic or causal contexts.

From a policy-making perspective, this research offers valuable guidance for updating national radiation safety regulations—especially in China, where current occupational exposure limits for the eye lens remain less stringent than international standards such as those of the ICRP. By providing a validated and evidence-based predictive tool, our findings support efforts to revise these limits toward more protective thresholds. Policymakers could mandate regular eye lens dose monitoring in high-risk environments like interventional radiology, ensuring timely implementation of protective measures. Moreover, this model offers a cost-effective solution for hospitals with limited resources, as it enables effective eye lens dose monitoring through machine-recorded cumulative exposure data, without necessitating full-body dosimetry systems. This makes high safety standards attainable across a broader range of healthcare facilities. Beyond national policy, this model could also contribute to the global standardization of radiation safety protocols. By offering a unified predictive framework, it facilitates cross-institutional and international comparisons of dose data, supporting benchmarking and fostering global collaboration to enhance the protection of healthcare workers.

Ensuring the effectiveness of radiation protection measures is essential for occupational safety. While this study provides a predictive model for eye lens dose estimation, comprehensive evaluation of protection effectiveness remains crucial. Our findings confirm a significant non-linear relationship between cumulative fluoroscopy dose and eye lens dose, suggesting that radiation risks increase disproportionately at higher exposure levels. This underscores the need to further investigate underlying factors such as radiation scattering, operator positioning, and procedural complexity. To enhance safety practices, real-time monitoring systems should be integrated into clinical workflows, enabling continuous assessment of radiation protection performance and timely interventions as needed. Future studies should explore the development of standardized evaluation methods to systematically assess and improve radiation protection strategies.

### Limitations

This study has several limitations. First, the sample size, though adequate for model development, was limited to 85 professionals from three hospitals. A larger and more diverse sample would enhance the model's generalizability. While key confounders such as hospital, procedure type, and operator role were accounted for, unmeasured factors such as individual radiation protection practices and equipment variability may influence dose distribution. Second, the quadratic polynomial regression model demonstrated significant predictive accuracy for cumulative fluoroscopy doses >20 Gy, but exhibited limited utility for doses ≤ 20 Gy. This discrepancy may arise from the dosimeters' minimum detectable level (MDL = 0.013 mSv), background radiation interference. Third, the validation was conducted in a single setting; broader validation across diverse hospitals and equipment is needed to ensure model robustness. Fourth, this study did not assess radiation energy and direction, as TLDs only provide cumulative dose measurements. Future studies could incorporate optically stimulated luminescence (OSL) dosimeters with filter-based spectral analysis to improve dose characterization and data interpretation. Finally, while the quadratic polynomial model performed well in high-dose scenarios, its inability to capture low-dose trends underscores the need for hybrid approaches, such as piecewise regression or machine learning models, to address the full dose spectrum. Future research should prioritize expanding the sample size and diversity, incorporating different equipment types, and investigating procedure-specific factors to enhance the generalizability of the findings.

## Conclusion

This study demonstrates a validated quadratic polynomial regression model capable of estimating eye lens dose based on cumulative fluoroscopy exposure in high-dose settings (>20 Gy). This model offers a practical tool to enhance occupational radiation protection and supports the need for stricter regulatory oversight of eye lens dose monitoring in China. Implementation of such predictive tools in clinical workflows may help align national practices with international standards, ultimately safeguarding the ocular health of interventional radiology professionals.

## Data Availability

The raw data supporting the conclusions of this article will be made available by the authors, without undue reservation.
