# Supplementary material for: Predictive modeling of eye lens dose in interventional radiology: a polynomial regression approach to cumulative fluoroscopy dose
Source: Front Public Health. 2025 May 20;13:1547101. doi: 10.3389/fpubh.2025.1547101 (PMC12129769; doi:10.3389/fpubh.2025.1547101)
Supplement: Supplementary file 1 [file Table_1.DOCX]

**Supplementary Material**

**Table S1 Dosimeter Specifications**

| **Parameter** | **Value/Description** |
| --- | --- |
| Manufacturer & Model | Beijing Haiyang Bochuang Technology Co., Ltd., SSCC-3 |
| Detection Type | *H_p_*​(3) (ICRU 1992) |
| Carrier Dimensions | 36 mm × 14 mm × 5 mm (external); 7.2 mm (internal diameter) |
| TLD Material & Dimensions | LiF: Mg, Cu, P; 4.5 mm diameter, 0.8 mm thickness |
| Headband Material | Polypropylene, adjustable length (520–920 mm) |
| Calibration Sources | N-100 X-rays, Cs-137 (662 keV) |

**
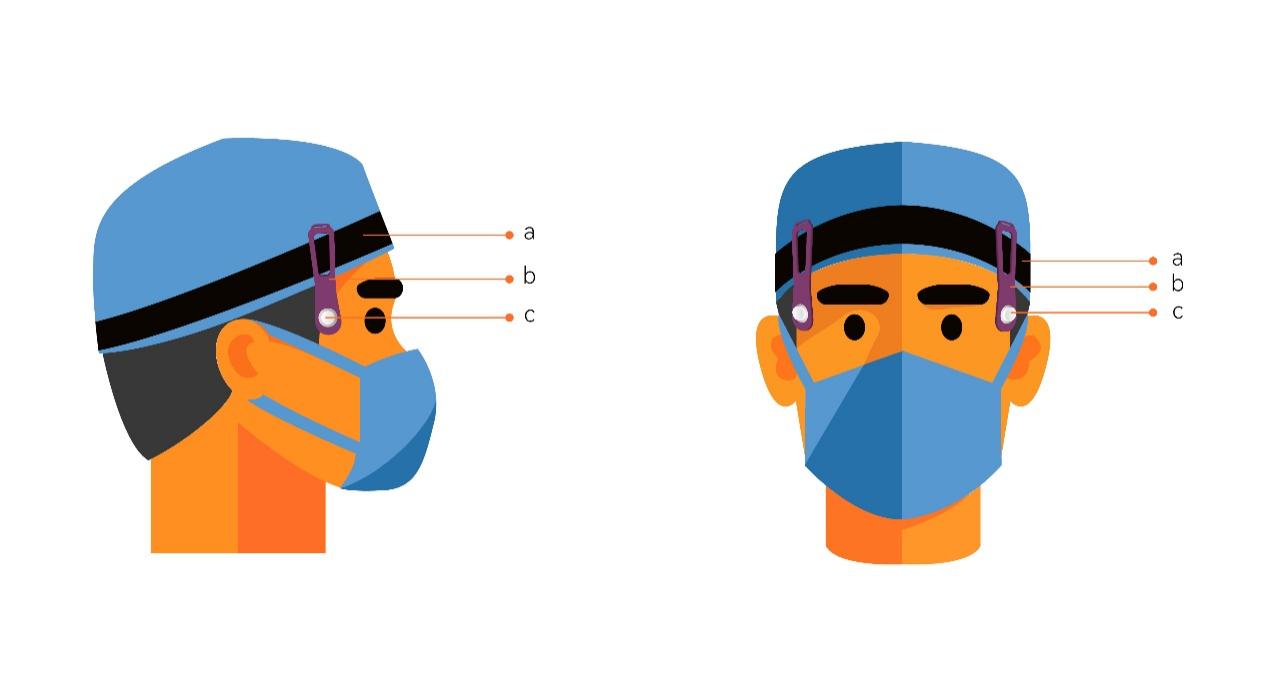
**

**Figure S1: The dosimeter placement**

Note: a: Adjustable headband; b: TLD carrier; c: TLD chip. The dosimeter was positioned on an adjustable headband at forehead level to measure eye lens exposure without protective eyewear, ensuring an accurate assessment of unshielded radiation exposure in real-world clinical conditions.

**Sample Size Calculation**

The sample size was calculated using statistical power analysis with the following parameters:

1. **Power:** Target power was set at 0.90.
2. **Alpha:** Significance level (α) was set at 0.05 for a two-tailed test.
3. **Correlation Coefficient:** The expected correlation coefficient was 0.512, based on results from a previous pilot survey.
4. **Simulations:** 5000 simulations were performed using a bivariate normal distribution to validate the sample size calculation.

Simulation results confirmed that a sample size of **40 participants** would achieve the target power of **0.90** with an actual power of **0.9054** (95% CI: 0.8973–0.9136). The actual alpha level was **0.0554**, within the 95% confidence interval (0.0491–0.0617), ensuring the reliability of the sample size calculation.

**Table S2 Descriptive Statistics of Fluoroscopy Dose and Eye Lens Dose**

| Variable | *M* | IQR (P_25_, P_75_) |
| --- | --- | --- |
| Cumulative Fluoroscopy Dose (Gy) | 5.087 | 9.041 (1.054, 10.095) |
| Left Eye Lens Dose (mSv) | 0.095 | 0.367 (0.056, 0.423) |
| Right Eye Lens Dose (mSv) | 0.096 | 0.299 (0.050, 0.349) |

**Note:** Displays the median and interquartile ranges for cumulative fluoroscopy and eye lens dose values.

**Table S3 Comparison of Median (IQR) Eye Lens Doses by Procedure Type**

| **Procedure Type** | **N** | **Left Eye [Median (Q1, Q3), mSv]** | **Right Eye [Median (Q1, Q3), mSv]** | **Z-value** | **P-value** |
| --- | --- | --- | --- | --- | --- |
| Cerebral Vascular | 11 | 0.074 (0.056, 0.190) | 0.080 (0.073, 0.245) | -0.445 | 0.657 |
| Thoracic Vascular | 10 | 0.075 (0.018, 0.373) | 0.048 (0.033, 0.307) | -0.051 | 0.959 |
| **Cardiac Vascular** | 37 | **0.097 (0.087, 0.494)** | 0.102 (0.070, 0.353) | **-2.406** | **0.016** |
| Peripheral Vascular | 11 | 0.099 (0.037, 1.634) | 0.131 (0.034, 0.841) | -0.445 | 0.657 |
| Lumbar | 15 | 0.065 (0.025, 0.349) | 0.094 (0.034, 0.349) | -0.682 | 0.496 |
| Other | 1 | – | – | – | – |
| **Total** | 85 | 0.095 (0.053, 0.423) | 0.096 (0.048, 0.349) | -1.593 | 0.111 |

**Notes:** Median (Q1, Q3) = Median with first and third quartiles. Bold values indicate statistically significant differences (p < 0.05).

**Model Selection and Rationale**

In this study, three statistical models were employed to investigate the relationship between cumulative fluoroscopy dose and eye lens dose: the General Linear Regression Model, the Restricted Cubic Spline (RCS) Model, and the Polynomial Regression Model. The following sections provide a detailed rationale for selecting the most suitable model based on various performance metrics and graphical representations.

**General Linear Regression Model**

The General Linear Regression Model assumes a simple linear relationship between cumulative fluoroscopy dose and eye lens dose. While this model is straightforward and interpretable, it has limitations when dealing with non-linear associations. In this study, the Adjusted R² values for the left and right eye dose predictions were relatively low (0.255 for the left eye and 0.142 for the right eye), indicating that this model explained only a small portion of the variance in eye lens doses. Additionally, the AIC and BIC values were higher compared to the other models, suggesting a weaker overall fit. For a visual representation of this model, see **Figure S2**.


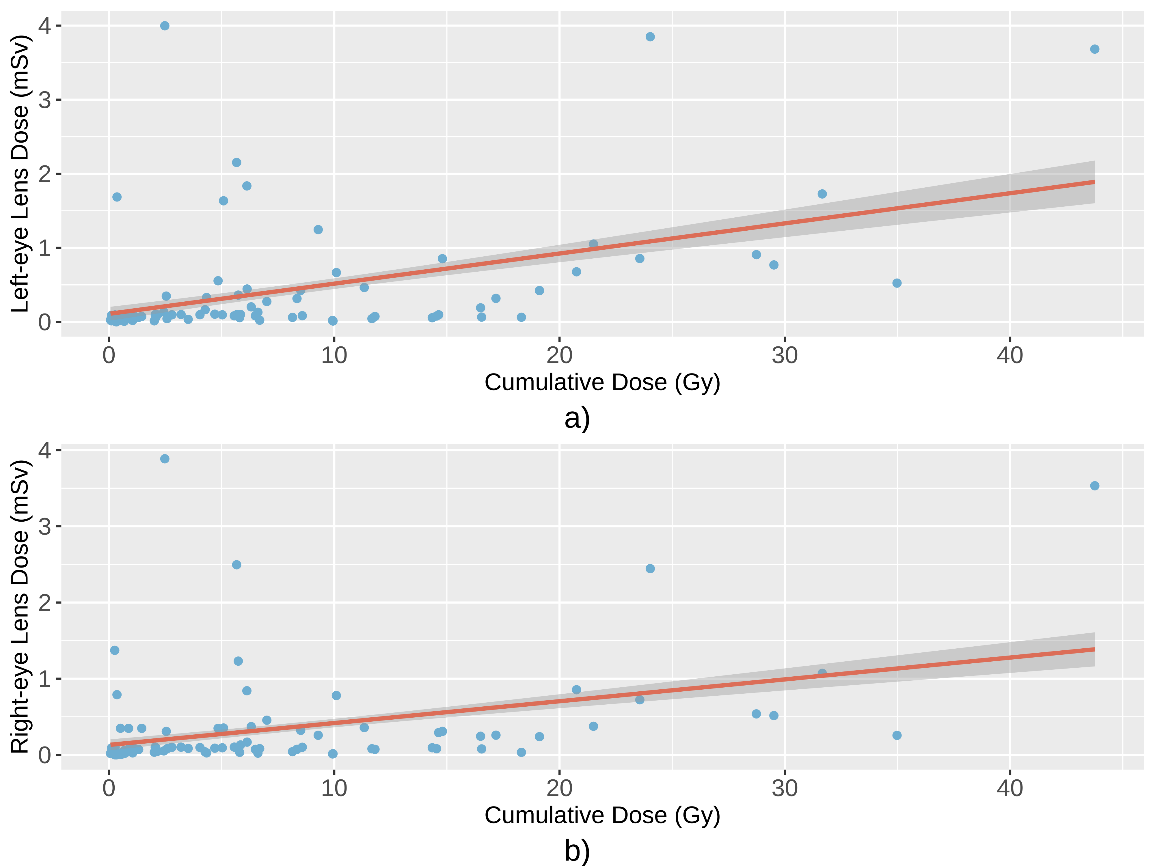


**Figure S2: General Linear Regression Model**

Note: Shows the linear relationship between cumulative fluoroscopy dose and eye lens dose. Confounders such as hospital and procedure type were controlled.

**Restricted Cubic Spline (RCS) Model**

The RCS Model introduces flexibility by allowing for non-linear relationships between cumulative fluoroscopy dose and eye lens dose. This model performed better than the General Linear Regression Model, as evidenced by higher Adjusted R² values (0.299 for the left eye and 0.233 for the right eye) and lower AIC and BIC values. The RCS Model captured subtle changes in the dose-response relationship, particularly at higher doses. However, while the RCS Model improved fit, the complexity of its interpretation compared to the Polynomial Regression Model was considered a limitation for practical applications. The RCS Model’s fit is visualized in **Figure S3**.


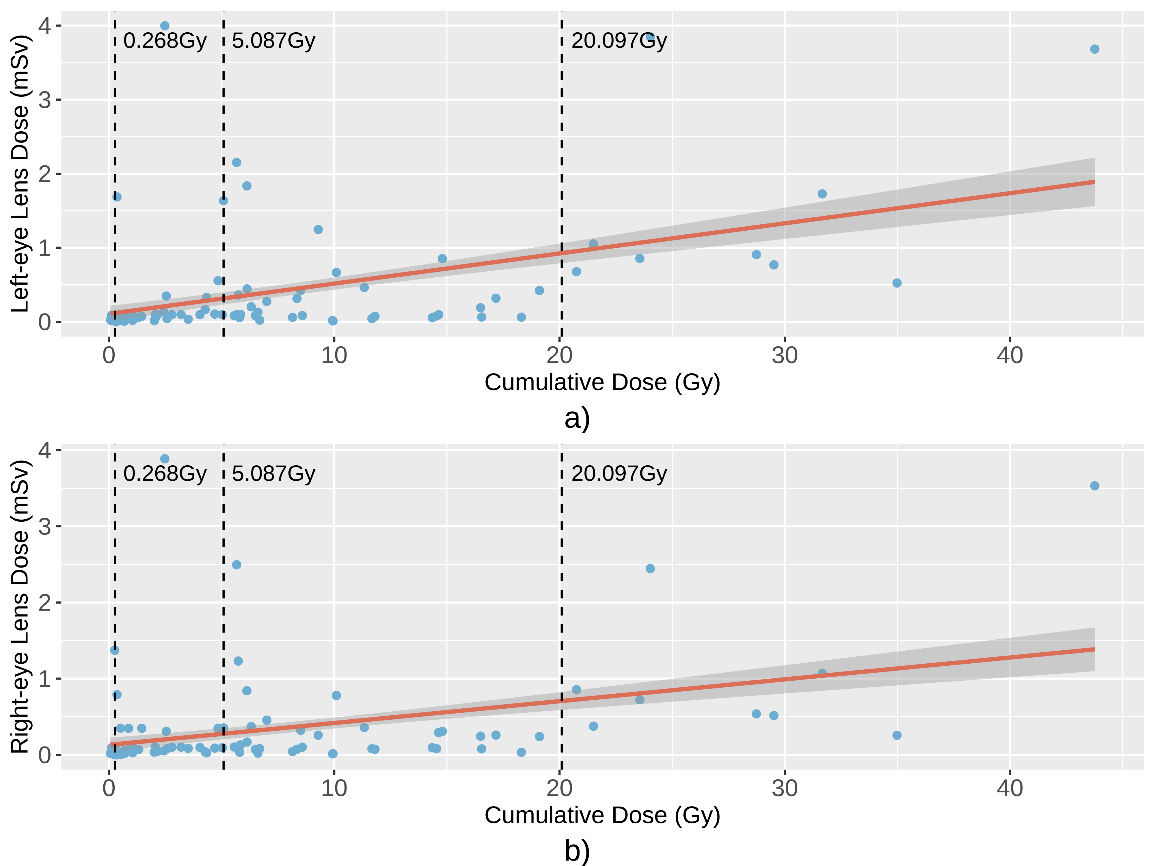


**Figure S3: Restricted Cubic Spline Model**

Note: The RCS Model fitted the dose-response relationship using the 10th, 50th, and 90th percentiles. Confounders such as hospital and procedure type were controlled.

**Polynomial Regression Model**

The Polynomial Regression Model emerged as the best-fitting model in this analysis, demonstrating the lowest AIC and BIC values and the highest prediction accuracy based on RMSE and MAE. This model provided a quadratic fit, capturing non-linear trends in the dose-response relationship while maintaining interpretability. The Adjusted R² values were 0.248 for the left eye and 0.201 for the right eye, slightly lower than the RCS Model, but the overall fit and predictive performance made this model the preferred choice for estimating eye lens dose based on cumulative fluoroscopy exposure.

The polynomial model equations for left and right eyes were as follows:

$$\begin{aligned} Left eye dose: Y_{left}=0.0013401X^{2}+0.2375347\#\left( 1 \right) \end{aligned}$$

$$\begin{aligned} Right eye dose: Y_{right}=0.0010375X^{2}+0.2080989\#\left( 2 \right) \end{aligned}$$

The validation of this model further demonstrated its robustness, with 79.57% of left eye dose estimates and 75.00% of right eye dose estimates falling within the model's 95% confidence interval. This validation confirms the model's practical applicability in estimating occupational radiation exposure for interventional radiologists. The quadratic fit of the Polynomial Regression Model is depicted in **Figure S4**.


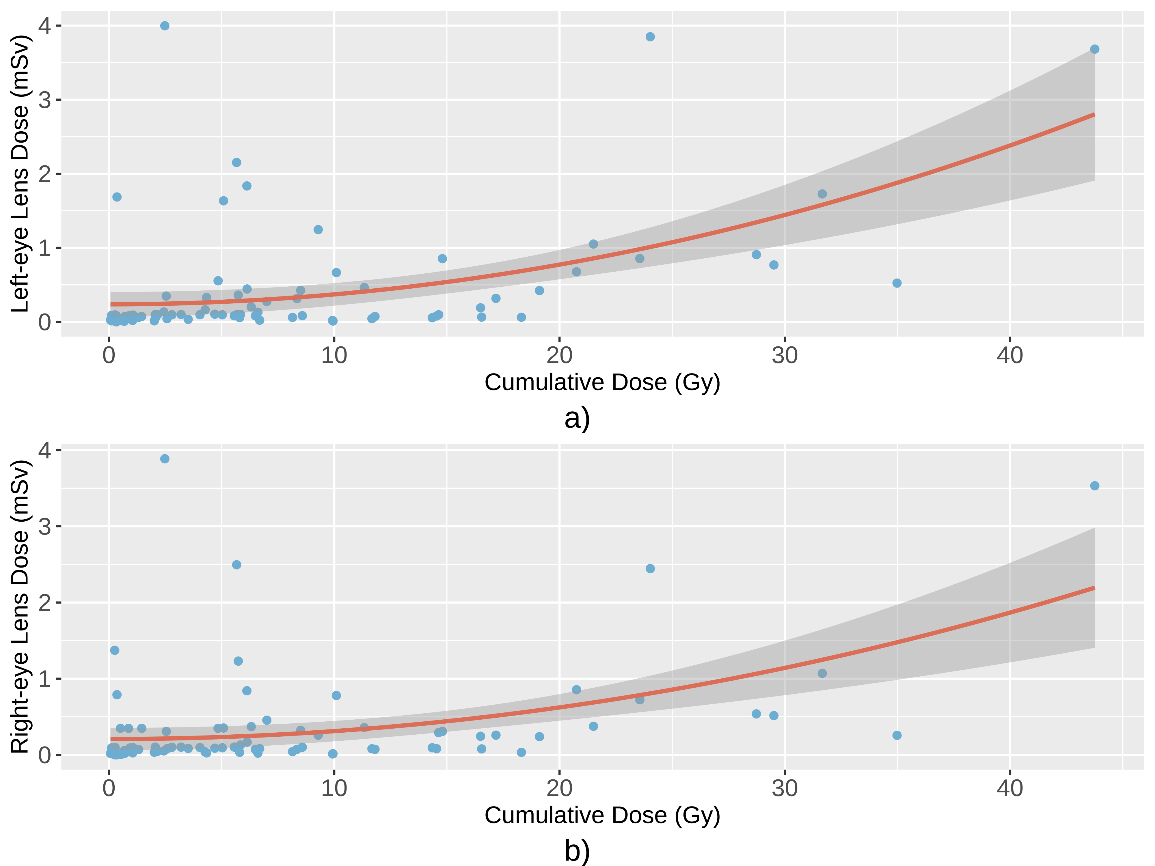


**Figure S4: Polynomial Regression Model**

Note: Depicts the quadratic dose-response relationship for both left and right eye lens doses. This model provides the best fit for the data.

**Residual Analysis for Model Validation**

Residual analysis was performed for the general linear, restricted cubic spline, and polynomial regression models to further evaluate their fit. **Figure S5** presents the residual plots for each model, focusing on the distribution of residuals around the horizontal dashed line. A well-fitting model should have residuals evenly distributed around the dashed line with no clear trends. As shown in **Figure S5**, the Polynomial model demonstrated the best distribution of residuals, supporting its selection as the most appropriate model.

In conclusion, the Polynomial Regression Model was selected as the most appropriate model for estimating eye lens dose based on cumulative fluoroscopy dose. Its superior fit, predictive accuracy, and validation results make it the most reliable model for use in clinical and occupational health settings. The model provides a practical tool for estimating eye lens dose and supports enhanced radiation protection measures for interventional radiology professionals.


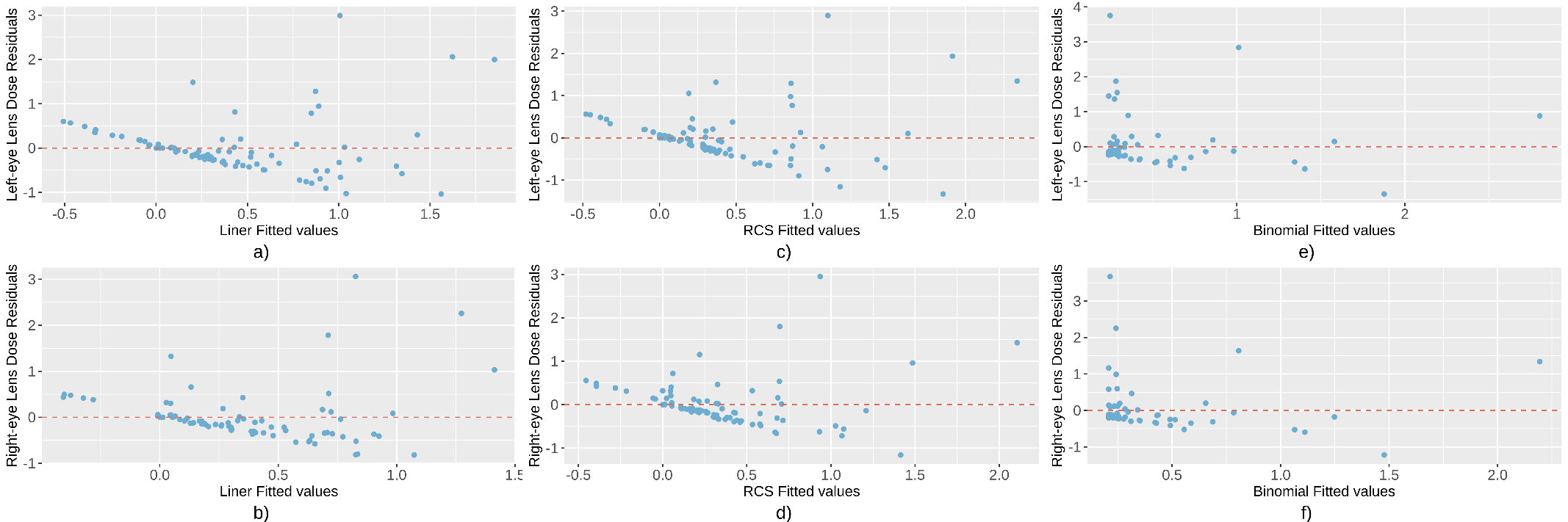


**Figure S5**, Residual Plots for General Linear, Restricted Cubic Spline, and Quadratic Polynomial Regression Models

Note: The residual plots examine the distribution of residuals across each model. Residuals should be evenly distributed around the horizontal dashed lines, indicating an acceptable fit if no noticeable trend is observed.

**Table S4 The quantified quality control framework**

| **Primary Indicator** | **Score** | **Secondary Indicator** | **Score** | **Tertiary Indicator** | **Score** | **Indicator Description** | **Scoring Criteria and Rules** | **Evaluation Method** | **Data Source** | **Points Earned** | **Calculation** |
| --- | --- | --- | --- | --- | --- | --- | --- | --- | --- | --- | --- |
| **I. Quantitative Indicators (10 points)** | | | | | | | | | | | |
| 1. Quantity | 10 | 1.1 Number of Interventional Procedures Monitored | 10 | / | / | Number of eligible personnel in the jurisdiction | Score (s) = (Actual number included) / Eligible personnel × 10 points | Evaluated based on the number of radiation workers by occupational category in each institution. | Chongqing Occupational Disease Prevention and Control Integrated Management Information System |  |  |
| **II. Quality Indicators (80 points)** | | | | | | | | | | | |
| 1. Organizational Management | 20 | 1.1 On-site Consistency Check of Operating Rooms | 10 | 1.1.1 Facilities and Equipment | 5 | Verify whether the operating rooms and equipment meet monitoring requirements for interventional procedures. | On-site verification of compliance. 1 non-compliance item deducts 1 point until fully deducted. | Direct evaluation based on on-site quality control results. | Institutional records; Chongqing Occupational Disease Prevention and Control Integrated Management Information System |  |  |
|  |  |  |  | 1.1.2 Quality Management System | 5 | Verify normal operation of the hospital's quality management system. | On-site audit of organizational structure, resource allocation, internal/external quality management, and documentation. 1 non-compliance item deducts 1 point until fully deducted. | Direct evaluation based on on-site quality control records. | On-site quality control records |  |  |
|  |  | 1.2 Information Maintenance | 10 | / | 10 | Accuracy and timeliness of registered information for interventional operating rooms. | Deduct 2 points for each missing/incorrect entry (e.g., personnel, equipment, projects). Unregistered items receive zero points. | Direct evaluation based on institutional records. | Institutional records |  |  |
| 2. Monitoring Process and Quality | 60 | 2.1 Monitoring Process | 45 | 2.1.1 Individual Monitoring Quality Control | 45 | 1. Eye lens dosimeters must be worn before entering the operating room.  2. Separate dosimeters for left and right eyes (no mixing).  3. Proper placement without obstruction during surgery.  4. Secure storage post-surgery to prevent damage or exposure.  5. Accurate logging of dosimeter usage.  6. Primary surgeon: closest to radiation source; Secondary surgeon: to the right of the primary surgeon.  7. Registration forms must match personnel and roles per procedure.  8. Document use of protective equipment during surgery.  9. Specify units for different instruments (cross out unused options). | 1 non-compliance item deducts 5 points until fully deducted. | Supervised by operating room staff; random audits by project team. | On-site inspection |  |  |
|  |  | 2.2 Data Information | 15 | 2.2.1 Surgical Registration Form Compliance | 15 | 1. White sections: filled by surgical staff post-procedure.  2. Gray sections: filled by operating room post-procedure.  3. Header: filled by operating room administrators.  4. Unique codes for monitored personnel. | 1 non-compliance item deducts 5 points until fully deducted. | Supervised by operating room staff; random audits by project team. | On-site inspection |  |  |
| **III. Outcome Indicators (10 points)** | | | | | | | | | | | |
| 1. Eye Lens Dosimeter Calibration | 5 | / | 5 | / | 5 | Calibration of eye lens dosimeters (twice) | Deduct 2.5 points for each missing calibration; zero points if uncalibrated. | Review calibration certificates and procurement records. | Calibration certificates |  |  |
| 2. Third-party Verification | 5 | Third-party Verification | 5 | / | 5 | Consistency verification for 10% randomly selected dosimeters by third-party laboratory. | 1 non-compliance item deducts 5 points. | Data rejected if deviation exceeds 20%; retained if within 20% threshold. | Third-party vs. laboratory data comparison |  |  |
| **Total Score = Quantitative + Quality + Outcome Scores. Results below 80 points are rejected.** | | | | | | | | | | |  |

**Table S5 Validation of the Polynomial Regression Model**

| Number of Monitored Staff (Hospital D) | Monitoring Duration (Days) | Left Eye Lens Dose (mSv) | Right Eye Lens Dose (mSv) | Cumulative Fluoroscopy Dose (Gy) | % of Left Eye Dose Values within 95% CI | % of Right Eye Dose Values within 95% CI |
| --- | --- | --- | --- | --- | --- | --- |
| 28 | 40 | 0.100（0.080~0.349） | 0.102（0.072~0.349） | 4.876（2.268~7.037） | 79.57 | 75.00 |

Note: This table presents the results of validating the Polynomial regression model using 28 monitored interventional radiologists from Hospital D over a 40-day period. The percentages show the proportion of actual eye lens dose values (both left and right eyes) that fell within the 95% confidence interval predicted by the model, indicating its accuracy.
